# Surgical Management of Arterial Injuries Within the Forearm: A Systematic Review

**DOI:** 10.7759/cureus.107559

**Published:** 2026-04-22

**Authors:** Chloe A Hanrahan, Jonathan Skurok, Emmet McKeown

**Affiliations:** 1 General Surgery, Causeway Hospital, Derry, GBR; 2 Emergency Medicine, Northumbria Specialist Emergency Care Hospital, Newcastle upon Tyne, GBR; 3 Emergency Medicine, Gloucestershire Royal Hospital, Gloucester, GBR

**Keywords:** artery, forearm, injury, ligate, patency, radial, repair, systematic review, ulnar, vessel

## Abstract

Traumatic injuries to the forearm often involve damage to the radial or ulnar arteries. In isolated single vessel injuries whereby the hand remains perfused, the surgeon is presented with a dilemma: to repair or ligate? This systematic review aims to compare the outcomes between patients who underwent surgical repair or ligation of vessels (PROSPERO: CRD42022311323). A systematic electronic search of Medline, Embase, CINAHL, PubMed, and Cochrane CENTRAL databases was conducted (March 11, 2022). All surgical comparative studies in adults sustaining a single forearm vessel injury with a minimum follow-up period of 12 months were included, evaluated using the Risk Of Bias In Non-randomized Studies of Interventions (ROBINS-I) and Critical Appraisal Skills Programme (CASP) tools. Three studies, totalling 80 patients, were included. Pooled long-term patency was 76% (48/63) across both radial and ulnar arterial repair. One study reported that ligated vessels showed a significant reduction in bone mineral density. Another study reported a significantly reduced lean muscle mass (p < 0.01) and strength following ligation. One study reported no difference in pain and cold intolerance between the ulnar artery ligation and repair groups. There is limited high-quality evidence available and significant heterogeneity within the reported outcomes. Our review suggests that repairing single vessel injuries is associated with improved outcomes; however, further high-quality studies on the topic are needed.

## Introduction and background

Vascular injuries to the forearm can present predominantly in the trauma setting, but also because of iatrogenic complications in theatre. They will involve damage to the radial, ulnar, or both arteries concurrently. Upon encountering such injuries, the surgeon must decide whether to repair the injured vessel or ligate it. Currently, in the United Kingdom, there are no guidelines for managing these patients despite the reality that vessel injury to the upper limb represents less than half of all vascular injuries [1]. The hand is unique in that it benefits from a dual perfusion system via the palmar arches. Consequently, if one vessel is injured, it is widely appreciated that the second vessel can maintain perfusion to the hand. Thus, ligation of the injured artery is a valid treatment option that will not result in hand ischaemia if collateral circulation stemming from the uninjured artery is intact. Vessel patency rates following surgical repair of forearm vessels have been documented as being over 50% in the literature [2]. Complications following vascular injury include cold sensitivity, pain, claudication, and weakness of the hand. Damage to nerves proximal to the site of injury can contribute to symptoms of cold sensitivity [3]. Additionally, it has been reported that cold sensitivity can present as an inevitable complication regardless of re-established blood flow through the affected vessel [4]. Complications such as nerve damage, cold sensitivity, and pain are important outcome measures to consider as they can have serious implications with regard to work, leisure, and overall quality of life. The aim of this systematic review is to review the available literature that investigates the reported outcomes of participants who have experienced injury to, and subsequent repair or ligation of, the radial and/or ulnar artery. By compiling the available evidence surrounding this surgical dilemma, we aim to identify the optimal management strategy that will best preserve the function of the forearm and hand.

## Review

Materials and methods

**Figure 1:**
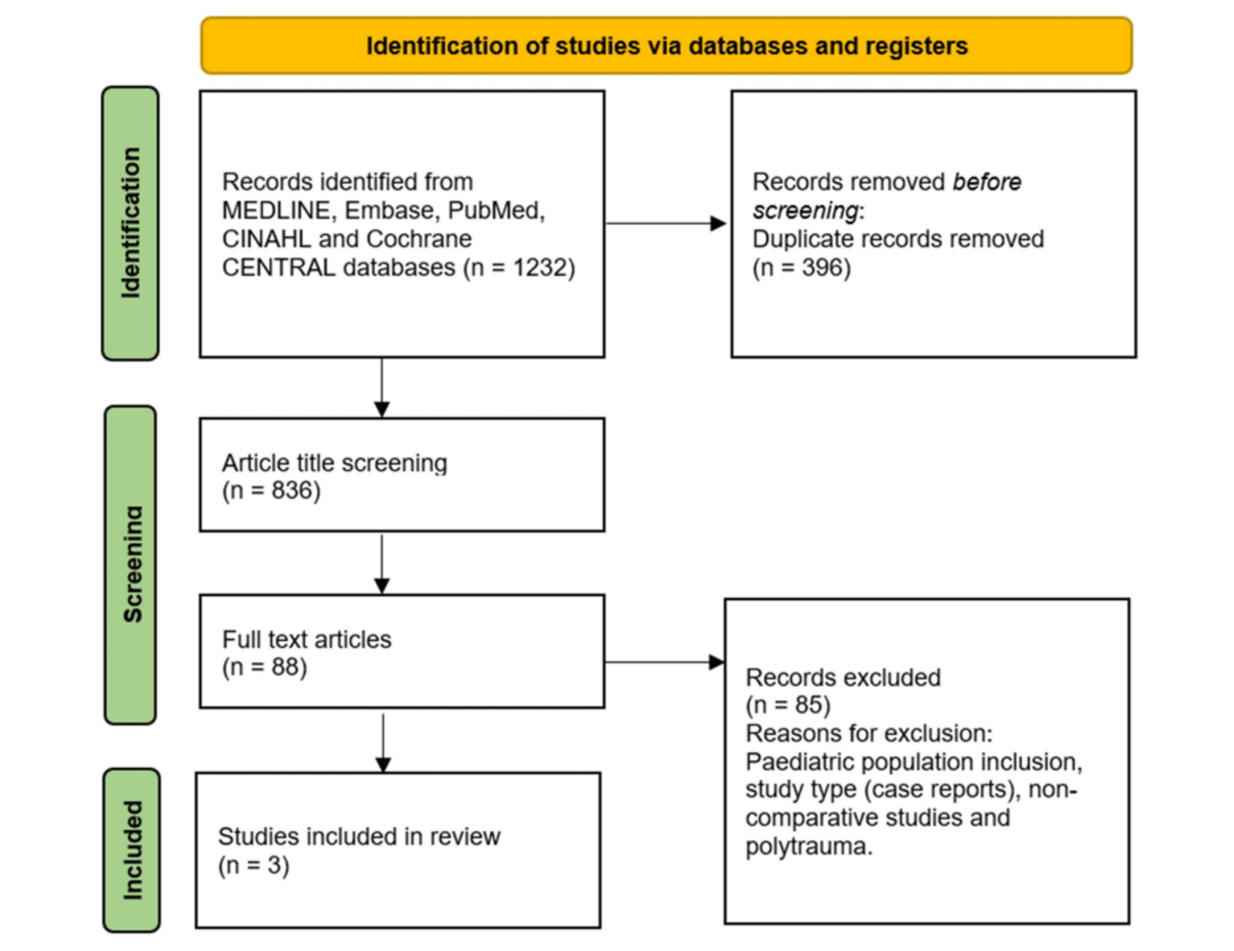
PRISMA figure showing the steps to choose the studies for systematic review PRISMA: Preferred Reporting Items for Systematic Reviews and Meta-Analyses

This systematic review was performed in accordance with the Preferred Reporting Items for Systematic reviews and Meta-Analyses (PRISMA) 2020 statement [5] and was prospectively registered on PROSPERO (Registration number: CRD42022311323). A clinical librarian performed an electronic search of MEDLINE, Embase, PubMed, CINAHL, and Cochrane CENTRAL databases for studies from their date of creation through March 11, 2022. Figure 1 summarises the search process in the form of a PRISMA flow diagram. No restrictions were applied to the electronic search. Reference lists of published reviews were also screened to identify any studies not yielded by the search strategy. The inclusion and exclusion criteria for this review are listed in Table 1. After removal of duplicates, the titles of studies identified from the search strategy were screened by two independent authors. Titles found to be relevant to the research question were compiled utilising the abstract screening tool, Rayyan (Rayyan Systems Inc., Cambridge, MA, USA) [6]. Studies that were selected from the abstract screening phase underwent a full-text screening phase. Where the inclusion of a study was contested by the two screening authors, the third author would settle any disagreements independently.

**Table 1:** Systematic review inclusion and exclusion criteria

Inclusion criteria	Exclusion criteria
Adult patients with single vessel injury to either ulnar or radial artery	Animal studies
Asymptomatic, perfused hand	Studies with insufficient follow-up data
Follow-up for outcomes for a minimum of 1 month	Patients with multiple injuries affecting upper extremity, e.g., palmar vessel injury and brachial artery injury
Case series	Patients with major nerve injuries involving the hand
	Patients without hand perfusion at the time of intervention

A novel Microsoft Excel data collection sheet (Microsoft Corp., Redmond, WA, USA) was created to be populated during the data extraction phase. In instances where data were missing from the publication, the authors were contacted via electronic mail to request supplementary data. A waiting time of 2-4 weeks was given for correspondence, after which the study was excluded. Patient and study characteristics are detailed in Tables 2, 3, respectively. The Cochrane Risk Of Bias In Non-randomized Studies of Interventions (ROBINS-I) tool was used to assess study risk of bias, as all included studies were case series [7]. The studies were assessed by two independent reviewers. Conflicts were resolved by consensus with a third author. Each study underwent quality assessment in line with the Critical Appraisal Skills Programme (CASP) protocol, which has been approved by the Cochrane Collaboration and permitted appraisal of the relevance and results of studies [8]. For contemporaneity, the original electronic search on MEDLINE, Embase, PubMed, CINAHL, and Cochrane CENTRAL databases was completed again in April 2026, which did not yield any salient studies for inclusion in this systematic review. 

**Table 2:** Study characteristics

Study	Study type	Aim	Participants	Age	Comparisons
Bassetto et al., 2010 [9]	Cohort study	To compare the effects of anastomosis/ligation on major arteries of the forearm (radial, ulnar, and both) after emergency surgery	Total = 53	Mean age = 45.7	End-end anastomosis = 43
			Male = 42	Range: 17-80 years	Vein graft = 2
			Female = 11		Ligation = 16
Zimmerman et al., 1994 [10]	Cohort study	To compare the recovery of ulnar artery occlusion after reconstruction or resection/ligation	Total = 14	Mean age = 47	Arterial reconstruction = 8
			Male = 12	Range: 31-72 years	Vein grafts = 6
			Female = 2		End-end anastomoses = 2
					Arterial resection = 6
Lannau et al., 2015 [11]	Cohort study	To assess the long-term patency of repaired radial and ulnar arteries	Total = 13	Mean age = 43	Arterial reconstruction = 13
			Male = 9	Range: unavailable	
			Female = 4		

**Table 3:** Study outcomes ^1^Patency assessed using arterial plethysmography, Doppler, and magnetic resonance angiography. ^2^Hand grip strength was quantified using the Dynatronic 100 ergometer (New Mechanics Pastorelli, Gallarate, VA, Italy). ^3^Muscle area and BMD were measured using peripheral quantitative computed tomography (pQCT). ^4^Forearm and hand lean mass measured using dual-energy X-ray absorptiometry (DEXA). ^5^Patency was assessed using Doppler. ^6^Digital Brachial Index (DBI) was calculated as the ratio of digital blood pressure to the simultaneous brachial artery pressure, measured using pneumoplethysmography. ^7^Pain frequency was measured as the number of days per week or month that pain was experienced, both before and after surgery. ^8^Functional limitation was assessed as the extent to which pain interfered with daily activities, both before and after surgery. ^9^Patency was assessed using Doppler. ^10^Hand grip and pinch strength were assessed using a dynamometer and pinch strength meter, respectively. ^11^Hand function questionnaires included the Michigan Hand Questionnaire (MHQ), the Cold Intolerance Symptom Severity (CISS) questionnaire, the Disabilities of the Arm, Shoulder, and Hand (DASH), and the modified CISS questionnaire. ^12^Pain frequency was assessed using the visual analogue scale.

Study	Outcomes	Follow-up duration	Main results
Bassetto et al., 2010 [9]	Vessel patency^1^	Mean: 11.2 ± 5.8 years. Range: 2-21 years	Patency 75%
	Hand grip strength^2^		Impaired BMD proximal (Δ –6%, p < 0.001) and distal (Δ –3%, p < 0.05) to the lesion, which correlated with vessel occlusion and trauma severity
	Muscle area and bone mineral density (BMD) at three sites and T scores of the distal radius^3^		Increased fracture risk following ligation/occlusion
	Forearm and hand lean mass^4^		1.5x higher risk of wrist fracture on the affected limb according to T scores
			Significant loss of lean mass and muscle strength in the affected limb, particularly in cases of occlusion of a major vessel
			Reduced hand grip strength in all patients
Zimmerman et al., 1994 [10]	Vessel patency^5^	Mean: 3.75 years	Patency 88%
	DBI of each digit (compared pre-operative to post-operative results)^6^. Change score calculated for small, ring, and index fingers		The difference in DBI between groups approached significance for the index finger (p = 0.056)
	Pain frequency^7^		No significant difference in cold and pain intolerance between the ligation and reconstruction groups
	Functional limitations due to pain^8^		
Lannau et al., 2015 [11]	Vessel patency^9^	Mean: 6 years	Patency 67%
	Hand grip strength and pinch strength^10^		
	Wrist range of motion		
	2-point discrimination of the thumb, index, and small finger		
	Hand function questionnaires^11^		No SS difference in CISS score, grip strength, pinch strength, 2-point discrimination, ranges of motion, pain between patent and non-patent groups for injured limb
	Pain frequency^12^		Clear trends in favour of the patent group for grip strength, 2-point discrimination (thumb and index finger), and the MHQ

Results

Study Selection

A total of 1,232 studies were identified after the database searches. 396 duplicate records were removed, leaving 836 studies that underwent title screening. Following abstract screening, 88 studies were selected for full-text screening, and 85 studies were excluded. There were various reasons for exclusion, including paediatric population inclusion, the study type (e.g., case reports), non-comparative studies, and studies with polytrauma. After full-text screening, three studies were included in this systematic review [9-11]. Inter-rater reliability between the two reviewers during full-text screening was assessed, and the two reviewers were deemed to be in substantial agreement (percentage agreement: 84%).

Study Characteristics

The characteristics of each of the three included studies are outlined in Table 2. All three of the included studies were cohort studies. The total number of participants was 80 (male, n = 63; female, n = 17). The overall mean age of participants ranged between 43 and 47 years, and the overall mean follow-up time was between three and 11 years. The overall mean age of participants was 46 years. All three studies reported on outcomes following arterial reconstruction [9-11]. Two of the studies reported the outcomes of arterial ligation [9,10]. There was heterogeneity amongst the outcome measures utilised to assess the efficacy of either repair or ligation of the injured vessel. All three studies assessed arterial patency using Doppler ultrasonography [9-11], and two studies assessed the effect of arterial reconstruction on hand grip [9,11]. One study assessed the effect of the intervention types on bone mineral density (BMD) and muscle area [9]. Another study compared the Digital Brachial Index (DBI) values of each digit between the repair and ligation groups and assessed the difference in cold and pain intolerance between these two groups [10].

Vessel Patency

Across the three studies, the pooled vessel patency was 76% (48/63) following ulnar and radial artery repair at follow-up using ultrasound imaging techniques. Zimmerman et al. compared the DBI pre-operatively and at follow-up and found that when the repair group was compared to the ligation group, the DBI change in the index finger of the reconstructed artery approached significance (p = 0.056).

BMD, Lean Muscle Mass, and Hand Grip Strength

Bassetto et al. measured the changes in BMD and subsequent fracture risk using dual-energy X-ray absorptiometry (DEXA) scans pre-operatively and following both interventions [9]. BMD was recorded at the proximal radius (cortical BMD), distal radius (trabecular BMD), and the second phalanx of the third finger (trabecular and cortical BMD). The study revealed that there was a statistically significant reduction in trabecular BMD at the distal radius in patients with non-patent (202 ± 43.4 vs. 214.6 ± 41.7 mg/cm^3^, p < 0.05) and ligated (208.6 ± 47.7 vs. 223.3 ± 34.3 mg/cm^3^, p < 0.05) arteries. Patients who underwent arterial ligation also had a significant reduction in cortical BMD (the reduction was recorded as Δ -5%, p < 0.05). Bassetto et al. also measured differences in BMD using peripheral quantitative computed tomography to study lean muscle mass changes [9]. A reduction in lean muscle mass was noted at the distal radius and at the second phalanx of the third finger in patients with ligated arteries (p < 0.05). Hand grip strength, corrected for hand dominance, was also assessed as a measure of functional outcome. A statistically significant reduction in hand grip was found in all affected limbs regardless of intervention type or patency (patent: Δ -16, p < 0.01; occluded: Δ -19%, p < 0.01; ligated: Δ -16%, p < 0.05) compared to the contralateral unaffected limb.

Cold and Pain Intolerance

Zimmerman et al. studied 14 participants, with six patients undergoing vessel ligation and eight undergoing vessel reconstruction [10]. The study reported no statistical significance in cold and pain symptoms between the ligation and reconstruction groups at follow-up.

Reporting Biases and Quality Assessment

The two reviewers independently analysed the three included studies for reporting bias using the ROBINS-I tool appropriate for cohort studies [7]. A third reviewer resolved any conflicts. One study had an overall serious risk of bias [9], whilst the other two studies had critical risks of bias [10,11]. Bias due to the selection of participants and the measurement of outcomes had the biggest influence on the overall risk of bias in the former study. Bias due to confounding factors had the greatest impact on overall risk in the latter two studies. Across all studies, a low risk of bias was reported regarding bias in the classification of interventions, bias due to missing data, and bias in the selection of the reported results.

The quality assessment of each study was guided by the CASP checklist [8]. Of the three studies, Zimmerman et al. obtained a CASP score of 17, the lowest of the three studies [10]. The score was influenced by deficiencies in identifying confounding factors, application of results to the local population, and implications for clinical practice. There was also some ambiguity around credibility and the precision of results. Lannau et al. received a slightly higher score of 19, with a deficiency pertaining to the implication of results for practice [11]. There was ambiguity around some areas, including recruitment, exposure measurement, confounding factors, follow-up, and result application. The study by Bassetto et al. was determined to be of high quality with a score of 23 out of a maximum of 28 [9]. Some weaker areas of the study include identification of confounding factors, credibility and application of results, and implications for practice.

Discussion

This systematic review analyses the available research undertaken to elicit the best surgical intervention for radial and ulnar artery injuries. The included studies sought to identify the efficacy of the repair intervention, which, when results were pooled, yielded a patency of 75% at the time of follow-up. This patency rate demonstrates an increase in repair efficacy compared to the widely accepted single vessel repair patency rate of greater than 50% [2]. This systematic review discusses different outcomes that have been measured to ascertain the benefits of each intervention type, including BMD, grip strength, change in muscle area, cold insensitivity, and pain insensitivity.

The study by Bassetto et al. identified a reduction in BMD in the distal radius associated with ligation of the affected vessel [9]. The result of reduced distal BMD following artery ligation compared to anastomosis may have long-term implications for bone health [9]. Preservation of distal BMD may help prevent the development of osteoporosis and the risk of fracture of the hand, even in the event of a minor insult to the bone. Bassetto et al. also identified a reduction in muscle area at two lower limb areas associated with vessel ligation [9]. In terms of pain and cold intolerance, neither surgical intervention demonstrated a significant impact on these outcomes [10]. The case was the same for grip strength [9]. With these findings in mind, the results from this systematic review may suggest that arterial repair is the superior management option. However, due to the absence of directly comparable outcomes across all studies, along with the quality and risk of bias assessment results of the included studies, it cannot be conclusively determined which intervention is optimal for arterial injury management.

The relationship between arterial patency and symptomology has been discussed in the literature for over 30 years. Some studies have shown that patients without nerve damage can become symptomatic as a result of lost patency following arterial repair [12]. Bacakoğlu et al. also described the various factors that can influence patency: from patient haemodynamics, e.g., pressure differences at both sides of the lesion, as described by Hagen-Poiseuille’s law [13], to hand dominance and type of anaesthesia used [12]. Other studies report no difference in cold and pain sensitivity between the patent and non-patent groups [11,14]. Furthermore, Lannau et al. found that following associated nerve injury repair, there was a statistically significant difference in cold sensitivity (Cold Intolerance Symptom Severity questionnaire score, p = 0.02) between the affected and healthy hands of the patients [11]. However, there is evidence to suggest that nerve regrowth can occur if the affected vessel is repaired, which may have implications for management [4,14]. The variation in results highlights the need for high-quality studies that can account for variables such as patency, concurrent nerve damage, and patient-reported symptoms.

The results of our review are somewhat in conflict with a similar review [15]. The pooled vessel patency rate was 68% for radial artery repairs and 65% for ulnar artery repairs [15], which is in alignment with the result in this review (76%). However, the difference in inclusion and exclusion criteria stringency allowed for analysis of 19 studies, and thus, they could compare the cold symptoms reported in 11 studies. Schippers et al. agree that more research is needed in this area to analyse the association between interventions and negative symptoms [15]. They do, however, advocate for the ligation management option as they concluded that there was no difference in cold sensitivity between ligated and repaired vessels [15]. Schippers et al. also consider the cost versus benefit of a cheaper procedure, given that vessel repair requires microsurgery [15]. Our systematic review has identified that there are more outcomes to be considered when making the decision between repair and ligation of the injured vessels, including a reduction in BMD and a change in muscle area, along with a hand grip strength reduction associated with both procedures [9]. Furthermore, more high-quality research is required to thoroughly investigate outcomes associated with both intervention types.

This systematic review has assessed multiple outcomes reported in each study to assess the impact of two surgical interventions. A broad search strategy without a time or language restriction was done on four databases, and reference lists were analysed to identify any relevant studies not previously found on initial searches. Strict inclusion criteria were used to mitigate the risk of confounding. An effort was made to contact the authors of studies where data were lacking from the original published articles. The process of screening, data compilation, risk of bias assessments, and quality assessments of studies was completed by two independent authors, with conflicts resolved by a third reviewer. The studies were shown to be of a medium to high quality as identified by the CASP tool [8].

Limitations

There were some limitations to this systematic review that we attempted to account for. Unfortunately, due to the lack of studies that fit within our inclusion criteria, the scope of this review was limited by a small sample size and variability of outcome reporting. The outcomes assessed are each useful in identifying the effects of the two intervention types; however, most of the outcomes were unique to each paper, such as BMD and changes to lean muscle mass. Due to heterogeneity in reporting outcomes and limited attempts to compare results, a meta-analysis was not performed. A severe risk of bias was found with one study included in this systematic review [9], and a critical risk of bias was associated with the other two studies [10,11], mainly due to bias associated with confounding factors. Variation in the quality of studies was also observed. Thus, the results assimilated in this review must be considered, with the risk of bias and quality of the studies accounted for.

## Conclusions

Our systematic review found that there is still significant ambiguity surrounding the best surgical management of injured ulnar and radial arteries of the forearm. The results of this systematic review suggest that arterial repair may offer the patient more physiological benefits with relation to preservation of BMD and muscle mass, compared to the option of ligating the vessel. The pooled arterial patency rate at follow-up was 76%, indicating that whilst there can be complications, such as thrombosis of the repaired artery at a later stage, the efficacy of arterial repair is higher than previously reported in the literature. Given the significant heterogeneity seen across the studies with regard to measuring outcomes following surgical interventions, more high-quality research is needed to ascertain which surgical management option offers the patient the best possible return of form and function following forearm injury. We recommend a multicentre, randomised control trial comparing the two types of surgical intervention using an array of outcome metrics and patient-reported symptom questionnaires.

## References

[1] Borman KR, Snyder WH, Weigelt JA (1984). Civilian arterial trauma of the upper extremity. An 11 year experience in 267 patients. Am J Surg.

[2] Gelberman RH, Nunley JA, Koman LA, Gould JS, Hergenroeder PT, MacClean CR, Urbaniak JR (1982). The results of radial and ulnar arterial repair in the forearm. Experience in three medical centers. J Bone Joint Surg Am.

[3] Ballard JL, Bunt TJ, Malone JM (1992). Management of small artery vascular trauma. Am J Surg.

[4] Aftabuddin M, Islam N, Jafar MA, Haque E, Alimuzzaman M (1995). Management of isolated radial or ulnar arteries at the forearm. J Trauma.

[5] Page MJ, McKenzie JE, Bossuyt PM (2021). The PRISMA 2020 statement: an updated guideline for reporting systematic reviews. BMJ.

[6] Ouzzani M, Hammady H, Fedorowicz Z, Elmagarmid A (2016). Rayyan-a web and mobile app for systematic reviews. Syst Rev.

[7] Sterne JA, Hernán MA, Reeves BC (2016). ROBINS-I: a tool for assessing risk of bias in non-randomised studies of interventions. BMJ.

[8] CASP-UK. 2022. CASP Critical Appraisal Skills Programme. [Online]. [Accessed (2022). CASP checklists. https://casp-uk.net/casp-tools-checklists/systematic-review-checklist/.

[9] Bassetto F, Zucchetto M, Vindigni V (2010). Traumatic musculoskeletal changes in forearm and hand after emergency vascular anastomosis or ligation. J Reconstr Microsurg.

[10] Zimmerman NB, Zimmerman SI, McClinton MA, Wilgis EF, Koontz CL, Buehner PT (1994). Long-term recovery following surgical treatment for ulnar artery occlusion. J Hand Surg Am.

[11] Lannau B, Bliley J, James IB (2015). Long-term patency of primary arterial repair and the modified cold intolerance symptom severity questionnaire. Plast Reconstr Surg Glob Open.

[12] Bacakoğlu A, Özkan MH, Coşkunol E, Özdemir O, Ekin A (2001). Multifactorial effects on the patency rates of forearm arterial repairs. Microsurgery.

[13] Simon AC, Flaud P, Levenson J (1990). Non-invasive evaluation of segmental pressure drop and resistance in large arteries in humans based on a Poiseuille model of intra-arterial velocity distribution. Cardiovasc Res.

[14] Johnson M, Ford M, Johansen K (1993). Radial or ulnar artery laceration. Repair or ligate?. Arch Surg.

[15] Schippers SM, Hajewski C, Glass NA, Caldwell L (2018). Single forearm vessel injury in a perfused hand: repair or ligate? A systematic review. Iowa Orthop J.

